# Multi-Scale Learning with Sparse Residual Network for Explainable Multi-Disease Diagnosis in OCT Images

**DOI:** 10.3390/bioengineering10111249

**Published:** 2023-10-26

**Authors:** Phuoc-Nguyen Bui, Duc-Tai Le, Junghyun Bum, Seongho Kim, Su Jeong Song, Hyunseung Choo

**Affiliations:** 1 Department of AI Systems Engineering, Sungkyunkwan University, Suwon 16419, Republic of Korea; phuocnguyen@skku.edu; 2 College of Computing and Informatics, Sungkyunkwan University, Suwon 16419, Republic of Korea; ldtai@skku.edu; 3 Sungkyun AI Research Institute, Sungkyunkwan University, Suwon 16419, Republic of Korea; bumjh@skku.edu; 4 Department of Ophthalmology, Kangbuk Samsung Hospital, School of Medicine, Sungkyunkwan University, Seoul 03181, Republic of Korea; n09072@gmail.com; 5 Biomedical Institute for Convergence, Sungkyunkwan University, Suwon 16419, Republic of Korea; 6 Department of Electrical and Computer Engineering, Sungkyunkwan University, Suwon 16419, Republic of Korea

**Keywords:** optical coherence tomography, medical image analysis, multi-disease diagnosis, multi-scale learning, residual network

## Abstract

In recent decades, medical imaging techniques have revolutionized the field of disease diagnosis, enabling healthcare professionals to noninvasively observe the internal structures of the human body. Among these techniques, optical coherence tomography (OCT) has emerged as a powerful and versatile tool that allows high-resolution, non-invasive, and real-time imaging of biological tissues. Deep learning algorithms have been successfully employed to detect and classify various retinal diseases in OCT images, enabling early diagnosis and treatment planning. However, existing deep learning algorithms are primarily designed for single-disease diagnosis, which limits their practical application in clinical settings where OCT images often contain symptoms of multiple diseases. In this paper, we propose an effective approach for multi-disease diagnosis in OCT images using a multi-scale learning (MSL) method and a sparse residual network (SRN). Specifically, the MSL method extracts and fuses useful features from images of different sizes to enhance the discriminative capability of a classifier and make the disease predictions interpretable. The SRN is a minimal residual network, where convolutional layers with large kernel sizes are replaced with multiple convolutional layers that have smaller kernel sizes, thereby reducing model complexity while achieving a performance similar to that of existing convolutional neural networks. The proposed multi-scale sparse residual network significantly outperforms existing methods, exhibiting 97.40% accuracy, 95.38% sensitivity, and 98.25% specificity. Experimental results show the potential of our method to improve explainable diagnosis systems for various eye diseases via visual discrimination.

## 1. Introduction

**Figure 1:**
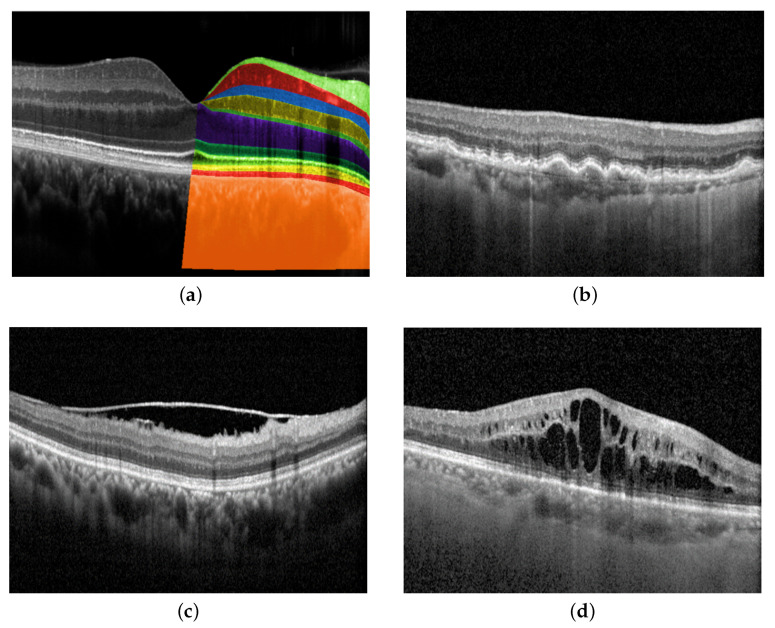
Retinal layers in OCT images and examples of eye diseases. (**a**) Retinal layers top to bottom: Nerve fiber layer, ganglion cell layer, inner plexiform layer, inner nuclear layer, outer plexiform layer, outer nuclear layer, external limiting membrane, ellipsoid zone, retinal pigment epithelial (RPE) interdigitation, RPE/Bruch’s membrane complex, choroid; (**b**) Age-related macular degeneration (AMD) is characterized by the build-up of drusen, which develops between the layers of RPE interdigitation and Bruch’s membrane complex; (**c**) Epiretinal membrane (ERM) is identified by the presence of a thin layer of scar tissue on the nerve fiber layer; (**d**) Macular edema (ME) refers to the accumulation of fluid in the macula, which is the central part of the retina from ganglion cell to outer nuclear layers.

In recent years, the application of deep learning in medical imaging has sparked a paradigm shift in the field of ophthalmology, heralding a new era of automated and precise diagnosis. Optical coherence tomography (OCT), a cornerstone of modern ophthalmic practice, offers unparalleled insights into the ocular anatomy and pathology. By harnessing the power of deep learning algorithms, OCT has transcended traditional manual analysis, enabling rapid, accurate, and standardized diagnoses that hold the promise of transforming patient care. An OCT image shows each layer of the retina at a high resolution, as described in Figure 1a. By interpreting OCT images, ophthalmologists are able to detect changes in the structure of the eye and investigate many pathologies, such as age-related macular degeneration (AMD), epiretinal membrane (ERM), and macular edema (ME), as depicted in Figure 1b, Figure 1c, and Figure 1d, respectively. Disease diagnosis in the early stages plays an important role in preventing vision loss.

Previous studies on automatic diagnostic development can be categorized into feature-based and deep learning-based methods. Feature-based methods typically adopt image processing techniques such as histograms of oriented gradient (HOG) [1], linear binary patterns (LBP) [2], and scale-invariant feature transform (SIFT) [3] to extract features for the final classifier. Although these methods have achieved promising results in situations where labeled data is scarce or computational resources are limited, they do not capture all relevant information in OCT images because of their limited representation capability, which reduces diagnostic accuracy. Another challenge is that the choice of feature extraction methods requires domain-specific expertise, which makes it difficult for non-experts to develop effective classifiers [4,5].

Deep learning-based methods have emerged as a popular approach for disease diagnosis in OCT images because of their ability to learn complex features directly from raw data. These models have shown state-of-the-art performance [5,6,7] in disease classification, demonstrating analytical capabilities corresponding to the diagnostic accuracy and sensitivity of ophthalmologists. Transfer learning is a commonly used approach in deep learning-based methods for eye disease classification using OCT images, which involves fine-tuning a pre-trained model on a smaller labeled dataset. However, deploying pre-trained models in practical applications is challenging because of their large number of parameters and high computational requirements [5]. At the same time, researchers have attempted to improve the performance by incorporating multi-scale features [8,9] and additional information such as the region of interest [10] and disease symptoms [11]. As a result, the reported approaches require considerable computational resources and effort to design the model and extract the necessary information [5].

Despite significant progress in the use of OCT for the diagnosis and management of retinal diseases, the current classification methods still have limitations. A prominent challenge is the need to address patients who may concurrently present with multiple diseases. Notably, many existing studies focus only on a single disease, with a specific focus on AMD [9] and ME [2]. On the other hand, some studies have made attempts at classifying multiple diseases but limit their data to images containing only a single disease [5,6,7], making it less practical for real-world applications. To the best of our knowledge, the largest and most common OCT dataset is OCT2017 [6], which contains 83,484 images with single-disease labels. The lack of a benchmark multi-label OCT dataset, where an image may contain signs from one or multiple diseases, limits the applications of the current diagnosis AI models in clinical environments.

**Figure 2:**
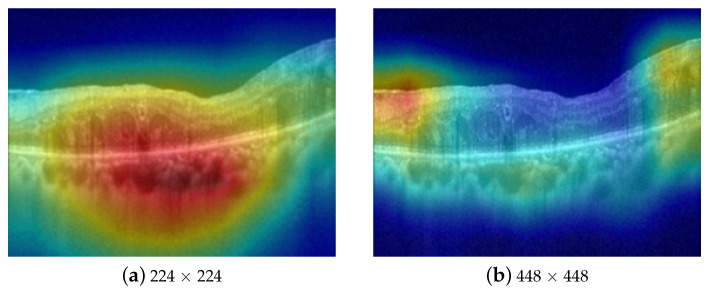
Attention maps of CNN on different scales of the same image. The model focuses on the accumulated fluid in (**a**) and the epiretinal membrane in (**b**), respectively.

In this paper, we collect and annotate a large-scale multi-label OCT data with approximately 33,000 images. Each image in this dataset is annotated with multiple diseases, including AMD, ERM, and ME. To perform multi-disease diagnosis using this extensive multi-label OCT dataset, we propose a simple yet effective multi-scale sparse residual network (MS-SRN) for multi-disease diagnosis in OCT images. First, the multi-scale learning (MSL) method effectively exploits the information from OCT images of different sizes to address the problem of varied disease lesions, improving the classification performance and enhancing interpretability, as shown in Figure 2. The MSL shows its effectiveness in improving the performance of different convolutional neural networks (CNNs). Second, the lightweight SRN consists of six convolutional blocks and employs residual learning for efficient learning. The proposed SRN uses only 6.1% of the learnable parameters compared with ResNet-101 but achieves similar performance in terms of all evaluation metrics. SRN is suitable for real-time applications because of its reduced number of parameters and reduced complexity. The combination of the MSL and SRN significantly outperforms other methods for multi-disease diagnosis in OCT images.

The main contributions of this paper are summarized as follows:

- We collected and annotated a large-scale multi-label OCT dataset with approximately 33,000 images, where each image is labeled as normal or abnormal with one or multiple diseases, including AMD, ERM, and ME.
- We propose a simple yet effective MSL method that fuses information from images of different sizes to improve classification performance and enhance visual interpretability. MSL shows its robustness when applied to different CNN architectures.
- The proposed SRN is a minimal residual network, where convolutional layers with large kernel sizes are replaced with multiple convolutional layers that have smaller kernel sizes, thereby reducing the model complexity while achieving better performance than the large kernel CNNs.
- Comprehensive experiments show that the proposed MS-SRN significantly outperforms the existing methods in terms of accuracy, sensitivity, and specificity. By combining MSL and SRN, we achieve superior performance while saving computational costs.

The remainder of this article is as follows: Section 2 summarizes related work. Section 3 formulates the problem and describes the proposed method and workflow in detail. Section 4 describes the datasets, implementation details, and evaluation metrics. Section 5 presents the results of the performance evaluation. Finally, Section 6 concludes the article.

## 2. Related Work

Recently, deep learning has brought about significant advancements in the interpretation of OCT images. This progress extends to various tasks, such as retinal layer and fluid segmentation [12,13,14,15], noise removal [16,17], image super-resolution [18,19], image generation [20], and disease classification [21,22]. For instance, in the context of retinal layer and fluid segmentation, researchers in [12] proposed a new convolutional neural architecture, namely RetiFluidNet, for multi-class retinal fluid segmentation. RetiFluidNet benefits from hierarchical representation learning of textural, contextual, and edge features via the attention mechanism [23]. On the other hand, OCT images are inevitably corrupted by speckle noise due to the coherence characteristics of scattered light. To enhance the OCT image quality, Zhou et al. [17] computed the weight of the non-local means using the deep features extracted by the self-supervised transformer and adopted the boosting strategy to realize an effective OCT image. In terms of disease classification, existing studies can be categorized into feature-based and deep learning-based methods.

**Feature-based methods**: Traditional machine learning approaches for automatic disease classification in OCT images consists of three main blocks: preprocessing, feature extraction, and classifier design. The preprocessing block, which involves techniques such as image denoising [24] and retinal flattening [3], is used to remove unwanted or redundant information from the raw input data and allows the model to extract meaningful information in the following stage. Next, feature descriptors such as histogram of oriented gradients [1], linear binary patterns [2], and scale-invariant feature transforms [3] are employed to manually extract features. Finally, the extracted features are fed into a classifier such as a random forest algorithm [25], a Bayesian classifier [23], or a support vector machine [2] to complete the classification. Although machine learning approaches have demonstrated promising results, they have several limitations. First, manual feature extraction is a time-consuming task that requires expertise, making it inefficient to build a large and comprehensive database. Furthermore, expert interpretations may differ, leading to results that may not be acceptable to other experts.

**Deep learning-based methods**: Previous studies [6,7] have employed pre-trained CNNs such as AlexNet [26] and InceptionNet [27] trained on ImageNet [28] and fine-tuned them using transfer learning. These models show accuracies of 97.1% and 96.1% on the OCT2017 dataset [6], respectively. However, the use of pre-trained networks with transfer learning has made the system complex due to the large number of parameters involved. Such networks are generally unsuitable for real-time deployment. To address this issue, Sunija et al. [5] proposed a lightweight CNN called OCTNet that achieves state-of-the-art (SOTA) performance with 99.6% accuracy on the OCT2017 dataset.

Multi-scale learning is another approach for disease classification in OCT images. Thomas et al. [9] proposed a multi-scale CNN with seven convolutional layers, allowing the network to detect a large number of local structures with different filter sizes to classify normal vs. AMD images, whereas Saman et al. [4] introduced a multi-scale CNN based on the feature pyramid network structure for single-disease multi-class classification. On the other hand, V. Das et al. [8] proposed a multi-scale deep feature fusion approach using four CNNs, which increases the inference time and computational complexity. The limitation of these methods is that they require a sophisticated model design and are not effective in challenging tasks, including multi-disease classification.

Attention-based methods have also been explored for disease classification using OCT images. For example, Fang et al. [11] demonstrated that detected macular lesion information can guide the network to focus on discriminative features and ignore insignificant information. However, their approach utilizes two separate networks, including a lesion detection network and a lesion-aware convolutional neural network, which increases computational complexity. Similarly, Huang et al. [10] used ReLayNet [29] for retinal layer segmentation and then employed a layer-guided convolutional neural network (LGCNN) to integrate the extracted information for classification. However, these methods are specific to eye diseases whose symptoms are easily detected, and their performances are significantly affected by the quality of the extracted information [11].

## 3. Method

### 3.1. Multi-Scale Learning Method

**Figure 3:**
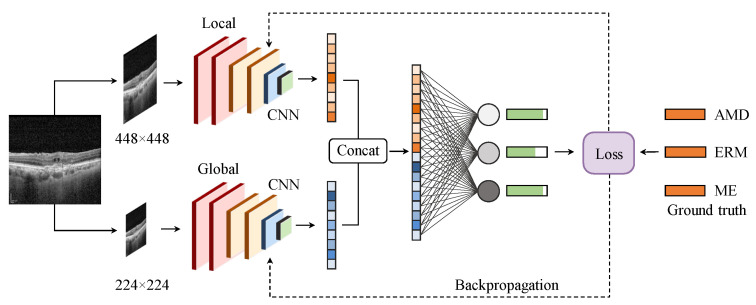
The proposed multi-scale learning method. Extracted features from the local (**top**) and global (**bottom**) branches are concatenated before feeding to a fully connected layer to produce the output.

Retinal diseases, such as ERM and ME, have lesions that come in various sizes, shapes, and orientations. For instance, intra-retinal fluid accumulation in ME is observed on a coarse scale due to its distinct, homogeneous texture in the retinal layers, as described in Figure 2a, whereas symptoms of ERM disease are quite small (see Figure 2b) requiring a finer scale for analysis. Fusing features from different image scales allows for capturing inter-scale variations, providing supplementary information for the classifier. Inspired by this observation, we propose an MSL method as depicted in Figure 3. The proposed learning method consists of two branches, namely local and global branches, where the former takes 448 × 448 images as input and the latter processes input images of size 224 × 224. A multi-label loss function $L_{ml}$ is used to compute the difference between the concatenated CNN output $\hat{y}$ and label *y* for the back-propagation process.

$$L_{ml}\left(\hat{y},y\right)=-\frac{1}{C}\sum_{i=1}^{C}y_{i}\log\left({\hat{y}}_{i}\right)+\left(1-y_{i}\right)\log\left(1-\hat{y_{i}}\right)$$

where *C* denotes the number of classes.

### 3.2. Sparse Residual Network (SRN)

Transfer learning, which fine-tunes pre-trained CNNs on different data, has played a significant role in the development of artificial intelligence-powered diagnosis tools and predictive models. These models are often well-trained on RGB images from the ImageNet dataset and contain a large number of parameters, which makes them impractically applicable in real-time environments. To address this problem, we designed a lightweight CNN, namely SRN, which has similar performance to existing CNNs but requires significantly fewer parameters and computational resources. Herein, we describe two design principles based on extensive experimentation with various architectures.

**Figure 4:**
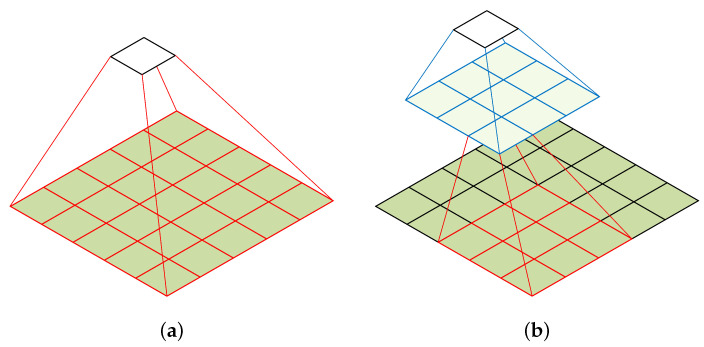
Factorization of a convolutional layer [27]. (**a**) 5 × 5 convolution. (**b**) Two-layer 3 × 3 convolution.

**Factorization of a convolutional layer**: A convolutional layer with a large kernel size is replaced with multiple convolutional layers that have smaller kernel sizes to reduce the number of parameters. Convolutional layers with a large kernel size are suitable for extracting high-level features such as shapes and patterns, which is critical in developing classification models for medical data. However, it also results in increased computational complexity and the loss of fine-grained details in the input. For example, a 5 × 5 convolution with n filters is 25/9 = 2.78 times more computationally expensive than a 3 × 3 convolution with the same number of filters. Inspired by [27], we discuss whether a 5 × 5 convolution could be replaced by a multi-layer network with fewer parameters but with the same input size and output depth as depicted in Figure 4.

To reduce the number of parameters in CNNs, we replace a single convolutional layer with a large kernel size with a multi-layer convolutional architecture that uses small kernel sizes. From the computation graph of the 5 × 5 convolution, each output is a small, fully connected network sliding over the 5 × 5 tiles of the input, as shown in Figure 4a. To exploit translation invariance and reduce the number of parameters, we replace the fully connected component with a two-layer convolutional architecture. The first layer is a 3 × 3 convolution, and the second layer is a 3 × 3 convolution applied to the 3 × 3 output grid of the first layer, as shown in Figure 4b. By sliding this small network over the input activation grid, we replace the 5 × 5 convolution with two layers of 3 × 3 convolution. This approach reduces the number of parameters in the model and is less computationally expensive. For example, a 5 × 5 convolution with n filters is 25/18 = 1.39 times more computationally expensive than a two-layer 3 × 3 convolution with the same number of filters.

**Residual learning**: Training deep neural networks (DNNs) can be challenging due to the problem of degradation. Degradation refers to the phenomenon in which the performance of very deep networks decreases as the network depth increases, even when a larger number of parameters are available to learn from the data. This occurs because, as the network becomes deeper, information from the input data can gradually vanish in the intermediate layers, resulting in a vanishing gradient problem. To overcome this challenge, residual learning [30] has been introduced as a technique for training DNNs. Instead of learning the direct mapping from input to output, residual learning focuses on learning the residual mapping, which represents the difference between the desired output and intermediate representations. Residual learning is implemented using residual blocks, which consist of convolutional and activation layers. These blocks use skip connections, where the input is added to the output, allowing the network to effectively capture the residual information.

**Figure 5:**
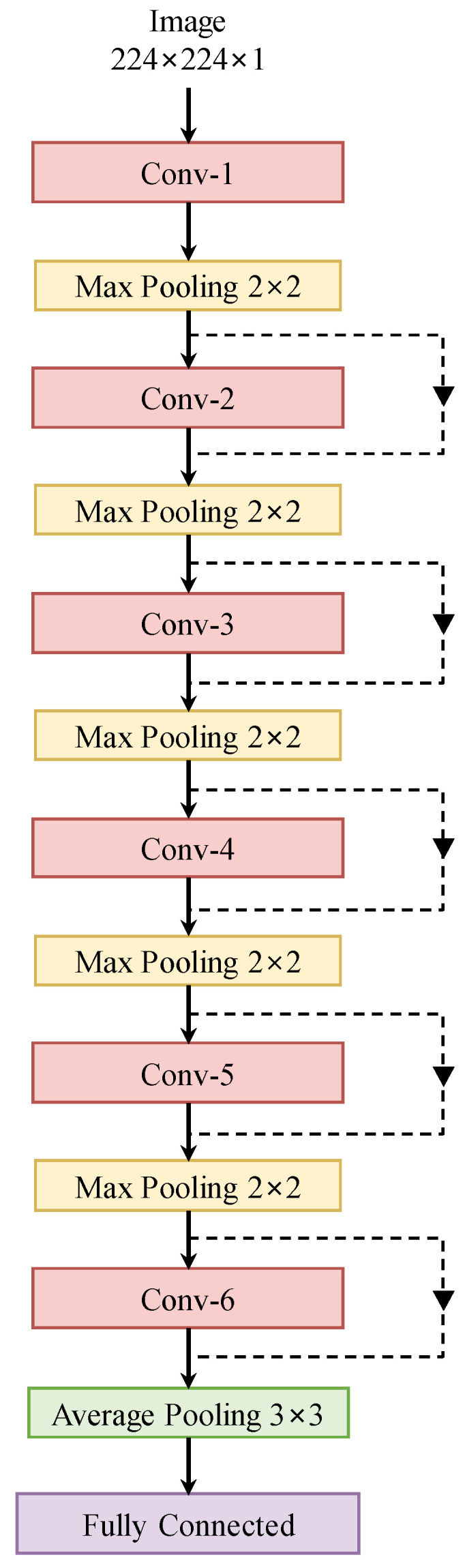
The proposed sparse residual network.

The proposed architecture is shown in Figure 5. We have six convolutional blocks, each of which is followed by a 2 × 2 max-pooling operation. Finally, an average pooling layer follows six blocks to encode the image into a vector of size 512 × 1. The feature vector is fed to a fully connected layer to produce the probability vector of the diseases. By modifying the number of layers in each convolutional block, we obtain multiple versions of SRN. In the proposed method for multi-disease diagnosis in OCT images, we combine 12-layer SRN with MSL.

## 4. Experiments

In this section, we first describe our OCT dataset and the metrics used for performance evaluation. We then provide the implementation details used to train our method.

**Figure 6:**
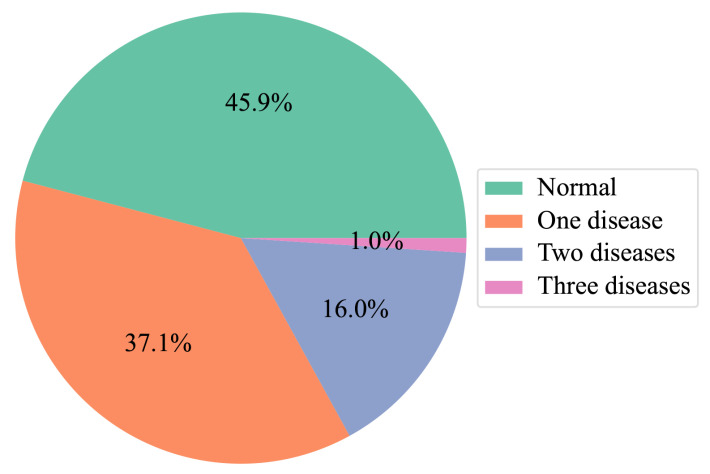
Distribution of images with multi-diseases.

**Dataset**: The largest and most common OCT dataset used in previous studies is OCT2017 [6], which contains 83,484 images with single-disease labels. Various studies have used this dataset to classify retinal pathologies using OCT images. However, the coexistence of multiple symptoms makes an accurate diagnosis a challenging task. We propose and collect a large OCT dataset for the multi-disease classification task, as presented in Table 1. High-quality OCT videos taken with Spectralis are collected and anonymized to protect the patient’s privacy. Each OCT video is split into frames, which are manually labeled by two ophthalmologists from Kangbuk Samsung Hospital (KBSMC). In particular, the labels annotated by a junior doctor are reviewed and verified by a senior doctor for accuracy and quality assurance. Figure 6 describes the distribution of our dataset.

**Evaluation metrics**: For each class, accuracy (Acc), sensitivity (Sen), and specificity (Spe) are used for performance evaluation. Based on the ophthalmologist’s opinion, we calculate the micro-average (μ-average) of each metric to have a more accurate representation of the overall performance. Micro-average accuracy is determined by aggregating the counts of true negatives, true positives, false negatives, and false positives across all classes and subsequently calculating the accuracy. Micro-average sensitivity is computed by summing up the counts of false negatives and true positives across all classes and then calculating the sensitivity. Micro-average specificity is derived by summing up the counts of false positives and true negatives across all classes and then calculating the specificity.

$$\begin{array}{cccc} \mathrm{Acc} & =\frac{TP+TN}{TP+FP+TN+FN} & \mu \mathrm{Acc} & =\frac{\sum_{i=1}^{C}\left(TP_{i}+TN_{i}\right)}{\sum_{i=1}^{C}\left(TP_{i}+FP_{i}+TN_{i}+FN_{i}\right)}\\ \mathrm{Sen} & =\frac{TP}{TP+FN} & \mu \mathrm{Sen} & =\frac{\sum_{i=1}^{C}TP_{i}}{\sum_{i=1}^{C}\left(TP_{i}+FN_{i}\right)}\\ \mathrm{Spe} & =\frac{TN}{TN+FP} & \mu \mathrm{Spe} & =\frac{\sum_{i=1}^{C}TN_{i}}{\sum_{i=1}^{C}\left(TN_{i}+FP_{i}\right)} \end{array}$$

where *C*, *TP*, *TN*, *FP*, and *FN* denote the number of classes, true positives, true negatives, false positives, and false negatives, respectively.

**Implementation details**: The entire dataset is split into a training set (80%) and a testing set (20%). We first resize the OCT images and then apply data augmentation techniques such as random rotation and horizontal/vertical flip. The proposed method is implemented using the Pytorch framework with random initialization weights on an NVIDIA A6000 GPU (48 GB). The batch size, learning rate, and the number of epochs are set to 64, 0.003, and 200, respectively. The stochastic gradient descent (SGD) optimizer is adopted with momentum and weight decay parameters set to 0.9 and 0.0001, respectively. All experiments are conducted with five different seeds, and then the mean and standard deviation values are calculated to produce solid results and ensure reproducibility.

## 5. Performance Evaluation

### 5.1. Comparison with Existing Works

In this section, we compare the proposed multi-scale sparse residual network (MS-SRN) with the existing work on multi-disease OCT image classification.

**Performance comparison with existing methods**: Existing methods are modified by replacing the softmax activation function with a sigmoid function for multi-label classification and trained in the same settings as our method. As presented in Table 2, the proposed MS-SRN outperforms other methods in terms of the micro-average value of all evaluation metrics. In particular, the results indicate the effectiveness of MS-SRN with up to 0.58% accuracy, 0.74% sensitivity, and 0.41% specificity improvement over transfer learning-based approaches [6,7,31]. Compared with the multi-scale-based approaches [8,9], the proposed method achieves superior performances with up to 7.07% accuracy, 12.79% sensitivity, and 2.84% specificity improvement. These results are verified by a student *t*-test as described in Table 3, in which a *p*-value of 0.05 or less is regarded as statistically significant.

**Figure 7:**
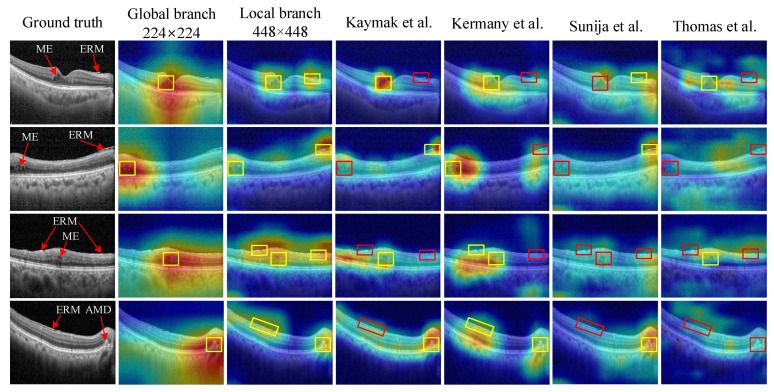
Heatmap-based interpretable inferences for disease detection. The proposed method successfully identifies disease-related areas in input images, as indicated by yellow boxes, while the other models [5,6,7,9] overlook such discriminative regions, as shown by red boxes.

Heatmaps derived from DNNs offer a visual representation of significant regions within an image, providing insights into the decision-making process of the network and enhancing the interpretability of a model. In our experiments, we utilize the Grad-CAM technique [34] to generate heatmaps for the proposed method as well as other approaches. These heatmaps provide in-depth explanations of the superior performance of the proposed method. As shown in Figure 7, our method, which combines features extracted from both the local and global branches, emphasizes the most relevant areas (highlighted by yellow boxes) to generate accurate predictions. In other words, the MSL method demonstrates the advantage of identifying lesions that may not be identified at a single scale but become distinguishable at higher or lower scales. Conversely, the other methods often fail to identify these critical regions (highlighted by red boxes), resulting in a decrease in diagnostic performance.

We also compare the complexity of the proposed model with other methods, including parameters, floating-point operations per second (FLOPs), and inference time per image. FLOPs and inference time are calculated on an NVIDIA GeForce RTX A6000 GPU using the PyTorch framework. The image sizes for each method are the same as those in their paper. As shown in Table 4, the InceptionV3 model employed in [6] has 21.79 M parameters, which is 4.21 times our method, resulting in a relatively prolonged inference time of 32.01 ms. Other CNN-based methods in [5,8,9] have fewer parameters and shorter inference times; their performances are significantly worse than our method, as shown in Table 2. Although these models are effective in single-disease diagnosis, as reported in their literature, they are not suitable for tackling more complicated tasks like multi-disease classification in OCT images. Compared with transformer-based methods [32,33], which have a large number of parameters due to the transformer structure with a global self-attention mechanism, the proposed MS-SRN has significantly fewer parameters and shorter inference times but still achieves superior diagnosis performance. These findings highlight that our method not only yields good results but also keeps reasonable computational costs.

### 5.2. Evaluation of Multi-Scale Learning Method and Sparse Residual Network

In this section, we first demonstrate the benefits of the MSL method for improving the performance of four CNNs. We then show the effectiveness of two principles used to design the proposed SRN.

**Generalization of the MSL method**: To verify the generalization ability of the proposed MSL method, we conduct experiments with different CNNs, including ResNet [30], VGGNet [35], OCTNet [5], and the proposed SRN. As presented in Table 5, the MSL method boosts CNNs performance compared with single-scale learning (SSL) for all testing models. Specifically, the MSL method has improved the accuracy of OCTNet by up to 0.46% accuracy, by up to 0.89% sensitivity, and by up to 0.28% specificity. The reason behind these improvements is that MSL captures more nuanced and fine-grained information as well as the underlying structure of the data. However, it requires more computational resources compared with SSL. Therefore, it is essential to find a trade-off between classification accuracy and computational burden.

**Factorization of a convolutional layer**: In our experiments, we compare a non-factorized network, which utilizes convolutional layers with large kernel sizes (e.g., 7 × 7 and 5 × 5), with its factorized counterpart created using the factorization technique. The factorized network only consists of convolutional layers with small kernel sizes (e.g., 3 × 3). The results show the effectiveness of this technique in reducing the number of parameters in convolutional layers and improving performance as well. As shown in Table 6, a 12-layer factorized network has fewer parameters but exhibits accuracy, sensitivity, and specificity higher by 0.36%, 0.46%, and 0.30% than the corresponding non-factorized counterpart. The reason behind this improvement is that using multiple convolutional layers with smaller kernel sizes enhances the nonlinearity of the network, which allows for more complex and expressive representations to be learned. These results highlight the efficacy of the factorization technique in reducing the number of parameters in DNNs, thereby making them more efficient and easier to train.

**Residual learning**: We conduct experiments to compare the proposed SRN with a plain network obtained by removing the residual connection from the original one. As shown in Table 7, the results demonstrate that the residual network consistently outperforms the plain one in terms of accuracy, sensitivity, and specificity. In particular, the 12-layer residual network yields 0.16% accuracy, 0.2% sensitivity, and 0.14% specificity higher than those of the corresponding plain network. This suggests that residual connections enable easier network optimization by addressing the vanishing gradient problem, allowing deeper architectures to be trained effectively to achieve better performance.

### 5.3. Ablation Studies

In this section, we first reported the results of experiments conducted to find the best configuration among variations of the SRN. We then show the effect of the choice of image size on the performance.

**Patient-level evaluation**: For multi-disease classification, instead of only evaluating the traditional disease category-based performance, the performance should also be evaluated by the patient/eye at the same time. For instance, if a person suffers from three diseases at the same time and the model detects only two, it should not be classified as correct. The only way to consider an OCT image to be categorized correctly is to detect all the diseases marked in that image. The accuracy is calculated by the number of correctly classified cases over the total number of cases. As presented in Table 8, the proposed method significantly outperforms the existing works with a *p*-value of 0.05.

**Variations of SRN**: We compare variations of SRN with three CNNs to investigate the effectiveness of the proposed architecture. By modifying the number of layers in each convolutional block, we obtain multiple versions of SRN as presented in Table 9. We compare the proposed SRN with other CNN architectures in both diagnosis performance and computational cost aspects. According to Table 10, the deeper the SRN, the better performance it achieves. Compared with other CNNs, the 8-layer SRN outperforms OCTNet with a similar number of parameters (1.86 M) in terms of all evaluation metrics, whereas the 12-layer SRN (2.59M) uses only 6.1% of learnable parameters compared with ResNet-101 but achieves similar performance. In terms of computational cost, the 12-layer SRN has a similar number of FLOPs with ResNet-18 and OCTNet but achieves superior diagnosis performance. These findings show that the SRN is suitable for real-time applications due to its reduced complexity.

**Effect of image size**: Extensive experiments are conducted with the proposed SRN to investigate the effect of image size on the training of DNNs. We run experiments with three image sizes, including 112 × 112, 224 × 224, and 448 × 448 along with their combinations as presented in Table 11. For the SSL method, the performance degrades when doubling the image size from 224 × 224 to 448 × 448, which means that increasing the image size does not always improve the classification performance. Simultaneously, the MSL method demonstrates the advantage of identifying lesions that are not recognized at a single scale but become distinguishable at higher or lower scales, which consistently improves the classification results and enhances the interpretability of the model.

## 6. Discussion

The proposed MS-SRN not only outperforms other methods in multi-disease classification on OCT, as presented in Table 2, but also provides insights into its performance, as illustrated in Figure 7. By combining information from images of different scales, the MSL method demonstrates the advantage of identifying lesions that may not be identified at a single scale but become distinguishable at higher or lower scales. Additionally, MSL is a general method that can be applied to other CNNs such as VGGNet, ResNet, and OCTNet (Table 4). Notably, SRN achieves a performance similar to that of ResNet while containing considerably fewer parameters (Table 9).

One limitation of our work is the simplicity of the proposed multi-label OCT dataset, which includes only three diseases and a normal class. Although it serves as a valuable pilot dataset for multi-disease diagnosis, it does not fully capture the complexity of the clinical scenarios where patients may present with multiple concurrent diseases. In our future work, we plan to expand the dataset to include a wider range of diseases, making it more representative and enhancing the model’s versatility for real-world medical cases. Furthermore, the current multi-scale learning method simply concatenates the model outputs from local and global branches to produce the prediction via a fully connected layer. Other forms of information fusion can be applied to further improve the performance of the MSL method. Additionally, an active learning-based method with doctor assistance [36] has been proven to improve the performance of diagnosis systems. In future work, we will train our method in an active learning manner with the help of ophthalmologists to improve its effectiveness and robustness.

## 7. Conclusions

In this paper, we construct and annotate large-scale multi-label OCT data with approximately 33,000 images with multi-disease labels. To perform multi-disease diagnosis on this dataset, we propose a simple yet effective approach, namely MS-SRN, for multi-disease diagnosis in OCT images. By capturing both local and global features in the input images of different sizes, the MSL method not only improves the performance but also enhances the interpretability of the CNNs via visual discrimination. Regarding the proposed SRN, we employ factorization and residual learning principles to reduce the complexity while achieving a performance similar to that of existing CNNs. In particular, a convolutional layer with a large kernel size is factorized by employing multiple convolutional layers that have small kernel sizes to reduce the number of parameters. Through extensive experiments on our multi-label OCT dataset, the proposed MS-SRN shows its effectiveness and significantly outperforms other models in terms of accuracy, sensitivity, and specificity. Our method has demonstrated the potential to improve the diagnosis and treatment of a wide range of eye diseases. Due to the reduced complexity, the proposed method is suitable for real-time applications, enabling efficient and timely decision-making in clinical settings. In future work, we will address the limitations of our work mentioned in the Discussion section.

## Figures and Tables

**Table 1:** Distribution of diseases in OCT dataset.

Class Name	Abbr.	# of Images
Age-related macular degeneration	AMD	7273
Epiretinal membrane	ERM	9272
Macular edema	ME	3597
Normal	Normal	12,818

**Table 2:** Quantitative comparisons of accuracy, sensitivity, and specificity with SOTAs. The best results are highlighted in **bold**. ↑ denotes the higher, the better.

Method	AMD	ERM	ME	Normal	μ-Average
Accuracy ↑					
Kaymak et al. [7]	96.86 ± 0.21	96.74 ± 0.11	97.43 ± 0.25	96.42 ± 0.19	96.82 ± 0.15
Kermany et al. [6]	**97.40 ± 0.12**	96.74 ± 0.09	97.79 ± 0.03	**97.20 ± 0.03**	97.28 ± 0.06
Li et al [31]	97.05 ± 0.06	96.54 ± 0.03	97.45 ± 0.07	96.57 ± 0.05	96.90 ± 0.04
Sunija et al. [5]	96.86 ± 0.18	96.18 ± 0.18	97.60 ± 0.09	96.28 ± 0.19	96.73 ± 0.12
Thomas et al. [9]	94.09 ± 0.20	91.73 ± 0.25	95.97 ± 0.18	91.69 ± 0.21	93.37± 0.23
V. Das et al. [8]	91.59 ± 0.12	87.87 ± 0.18	94.69 ± 0.11	87.19 ± 0.15	90.33± 0.13
Dosovitskiy et al. [32]	96.41 ± 0.14	95.47 ± 0.18	97.27 ± 0.05	95.48 ± 0.15	96.16 ± 0.15
Liu et al. [33]	97.17 ± 0.11	96.28 ± 0. 31	97.60 ± 0.07	96.67 ± 0.30	96.93 ± 0.17
Proposed MS-SRN	97.31 ± 0.17	**97.07 ± 0.07**	**98.03 ± 0.11**	**97.20 ± 0.06**	**97.40 ± 0.04**
Sensitivity ↑					
Kaymak et al. [7]	**93.44 ± 0.45**	94.57 ± 0.73	90.33 ± 0.81	96.61 ± 0.52	94.64 ± 0.19
Kermany et al. [6]	93.14 ± 0.41	94.34 ± 0.15	90.81 ± 0.61	**98.08 ± 0.09**	95.13 ± 0.11
Li et al. [31]	93.13 ± 0.35	94.06 ± 0.03	88.95 ± 0.49	97.41 ± 0.04	94.59 ± 0.07
Sunija et al. [5]	93.01 ± 0.44	93.34 ± 0.75	88.83 ± 0.99	97.03 ± 0.28	94.20 ± 0.13
Thomas et al. [9]	86.33 ± 0.35	86.82 ± 0.23	80.92 ± 0.18	93.16 ± 0.20	88.51 ± 0.21
V. Das et al. [8]	80.27 ± 0.15	79.56 ± 0.20	66.99 ± 0.14	90.57 ± 0.12	82.59± 0.17
Dosovitskiy et al. [32]	90.07 ± 0.14	91.82 ± 0.43	85.75 ± 0.68	97.19 ± 0.38	92.84 ± 0.35
Liu et al. [33]	93.06 ± 0.49	93.93 ± 0.40	90.44 ± 0.19	97.06 ± 0.72	94.57 ± 0.33
Proposed MS-SRN	93.35 ± 0.79	**95.25 ± 0.63**	**90.95 ± 0.81**	97.90 ± 0.23	**95.38 ± 0.14**
Specificity ↑					
Kaymak et al. [7]	98.08 ± 0.20	97.83 ± 0.29	98.62 ± 0.17	96.25 ± 0.22	97.84 ± 0.06
Kermany et al. [6]	**98.92 ± 0.15**	97.95 ± 0.10	98.82 ± 0.06	96.46 ± 0.12	98.18 ± 0.10
Li et al. [31]	98.45 ± 0.05	97.79 ± 0.03	98.70 ± 0.15	95.87 ± 0.12	97.87 ± 0.04
Sunija et al. [5]	98.23 ± 0.34	97.62 ± 0.25	98.89 ± 0.21	95.66 ± 0.24	97.79 ± 0.11
Thomas et al. [9]	96.87 ± 0.14	94.21 ± 0.31	98.19 ± 0.20	90.44 ± 0.19	95.41 ± 0.22
V. Das et al. [8]	95.63 ± 0.14	92.06 ± 0.25	98.77 ± 0.28	84.36 ± 0.11	96.73 ± 0.18
Dosovitskiy et al. [32]	98.67 ± 0.23	97.32 ± 0.13	98.97 ± 0.06	94.04 ± 0.08	97.55 ± 0.06
Liu et al. [33]	98.64 ± 0.09	97.47 ± 0.66	98.65 ± 0.08	96.34 ± 0.09	97.92 ± 0.12
Proposed MS-SRN	98.72 ± 0.21	**97.99 ± 0.23**	**99.08 ± 0.08**	**96.61 ± 0.14**	**98.25 ± 0.05**

AMD: age-related macular degeneration, ERM: epiretinal membrane, ME: macular edema.

**Table 3:** Statistical significance test on accuracy metric. A *p*-value of 0.05 or less indicates that the improvement of the proposed method over the comparison method is statistically significant.

Method	AMD	ERM	ME	Normal	μ-Average
Kaymak et al. [7]	<0.05	<0.05	<0.05	<0.05	<0.05
Kermany et al. [6]	0.43	<0.05	<0.05	0.32	<0.05
Li et al [31]	<0.05	<0.05	<0.05	<0.05	<0.05
Sunija et al. [5]	<0.05	<0.05	<0.05	<0.05	<0.05
Thomas et al. [9]	<0.05	<0.05	<0.05	<0.05	<0.05
V. Das et al. [8]	<0.05	<0.05	<0.05	<0.05	<0.05
Dosovitskiy et al. [32]	<0.05	<0.05	<0.05	<0.05	<0.05
Liu et al. [33]	<0.05	<0.05	<0.05	<0.05	<0.05

AMD: age-related macular degeneration, ERM: epiretinal membrane, ME: macular edema.

**Table 4:** Analysis of parameters, FLOPs, and inference time of ours and other methods. The best results are highlighted in **bold**. ↓ denotes the lower, the better.

Method	# Params ↓	FLOPs ↓	Inference Time ↓
Kaymak et al. [7]	57 M	0.71 G	2.96 ms
Kermany et al. [6]	21.79 M	5.74 G	32.01 ms
Li et al. [31]	134.27 M	15.48 G	6.44 ms
Sunija et al. [5]	1.86 M	1.16 G	3.33 ms
Thomas et al. [9]	1.35 M	0.05 G	**2.32 ms**
V. Das et al. [8]	**0.14 M**	**0.02 G**	2.51 ms
Dosovitskiy et al. [32]	87.42 M	4.42 G	43.69 ms
Liu et al. [33]	86.68 M	15.50 G	134.30 ms
Proposed MS-SRN	5.18 M	5.79 G	16.03 ms

**Table 5:** Effectiveness of multi-scale learning on different CNNs. The best results are highlighted in **bold**. ↑ denotes the higher, the better.

Method	ResNet	VGGNet	OCTNet	SRN
Accuracy ↑				
w/o MSL (224 × 224)	97.28 ± 0.11	96.68 ± 0.08	96.73 ± 0.12	97.28 ± 0.09
w/o MSL (448 × 448)	97.21 ± 0.10	96.66 ± 0.03	96.49 ± 0.11	97.22 ± 0.04
w/ MSL ($224^{2}$ + $448^{2}$)	**97.39 ± 0.04** *	**96.92 ± 0.14** *	**96.95 ± 0.10** *	**97.40 ± 0.04** *
Sensitivity ↑				
w/o MSL (224 × 224)	95.06 ± 0.25	**94.47 ± 0.07**	94.20 ± 0.13	95.00 ± 0.14
w/o MSL (448 × 448)	95.04 ± 0.18	94.13 ± 0.03	93.56 ± 0.17	94.88 ± 0.12
w/ MSL ($224^{2}$ + $448^{2}$)	**95.38 ± 0.08** *	94.43 ± 0.09	**94.45 ± 0.10** *	**95.38 ± 0.14** *
Specificity ↑				
w/o MSL (224 × 224)	98.21 ± 0.09	97.61 ± 0.11	97.79 ± 0.11	98.23 ± 0.05
w/o MSL (448 × 448)	98.11 ± 0.08	97.73 ± 0.04	97.72 ± 0.08	98.20 ± 0.07
w/ MSL ($224^{2}$ + $448^{2}$)	**98.23 ± 0.02**	**97.96 ± 0.16** *	**98.00 ± 0.03** *	**98.25 ± 0.05**

* indicates that the performance difference between multi-scale learning and single-scale learning is statistically significant with a *p*-value of 0.05.

**Table 6:** Effectiveness of convolutional factorization in sparse residual networks. The best results are highlighted in **bold**. ↑ denotes the higher, the better. ↓ denotes the lower, the better.

Network	# Params ↓	Accuracy ↑	Sensitivity ↑	Specificity ↑
	12-Layer	12-Layer	12-Layer	12-Layer
Non-factorized	2.60 M	96.92 ± 0.10	94.54 ± 0.19	97.93 ± 0.11
Factorized	**2.59 M**	**97.28 ± 0.09** *	**95.00 ± 0.14** *	**98.23 ± 0.05** *

* indicates that the performance difference between factorized and non-factorized networks is statistically significant of a *p*-value of 0.05.

**Table 7:** Effectiveness of residual learning in SRN. The best results are highlighted in **bold**. ↑ denotes the higher, the better. ↓ denotes the lower, the better.

Network	# Params ↓	Accuracy ↑	Sensitivity ↑	Specificity ↑
	12-Layer	12-Layer	12-Layer	12-Layer
Plain	**2.41 M**	97.12 ± 0.16	94.80 ± 0.06	98.09 ± 0.12
Residual	2.59 M	**97.28 ± 0.09** *	**95.00 ± 0.14** *	**98.23 ± 0.05** *

* indicates that the performance difference between residual and plain networks is statistically significant of a *p*-value of 0.05.

**Table 8:** Quantitative comparisons of patient-level accuracy with SOTAs. The best results are highlighted in **bold**. ↑ denotes the higher, the better. A *p*-value of 0.05 or less indicates that the improvement of the proposed method over the comparison methods is statistically significant.

Method	Accuracy ↑	*p*-Value
Kaymak et al. [7]	91.65 ± 0.13	<0.05
Kermany et al. [6]	92.45 ± 0.23	<0.05
Li et al [31]	91.65 ± 0.12	<0.05
Sunija et al. [5]	91.21 ± 0.29	<0.05
Thomas et al. [9]	83.11 ± 0.10	<0.05
V. Das et al. [8]	76.56 ± 0.10	<0.05
Dosovitskiy et al. [32]	89.73 ± 0.29	<0.05
Liu et al. [33]	91.53 ± 0.41	<0.05
Proposed MS-SRN	**92.82 ± 0.13**	-

**Table 9:** Detailed settings in SRN’s variations.

Block	Output Size	8-Layer	10-Layer	12-Layer
conv-1	112 × 112		7 × 7, 32	
conv-2	56 × 56	3 × 3, 32	3 × 3, 32	3 × 3, 32
		3 × 3, 32	3 × 3, 32	3 × 3, 32
			3 × 3, 32	3 × 3, 32
conv-3	28 × 28	3 × 3, 64	3 × 3, 64	3 × 3, 64
		3 × 3, 64	3 × 3, 64	3 × 3, 64
				3 × 3, 64
conv-4	14 × 14	3 × 3, 128	3 × 3, 128	3 × 3, 128
			3 × 3, 128	3 × 3, 128
conv-5	7 × 7	3 × 3, 256	3 × 3, 256	3 × 3, 256
				3 × 3, 256
conv-6	3 × 3	3 × 3, 512	3 × 3, 512	3 × 3, 512
classifier	1 × 1	3 × 3 average-pooling, fully-connected		
# params		1.80 M	1.96 M	2.59 M

**Table 10:** Performance comparison between SRN’s variations and other CNNs. The best results are highlighted in **bold**. ↑ denotes the higher, the better. ↓ denotes the lower, the better.

Network	# Params ↓	FLOPs ↓	Single-Scale Learning			Multi-Scale Learning		
			Accuracy ↑	Sensitivity ↑	Specificity ↑	Accuracy ↑	Sensitivity ↑	Specificity ↑
VGGNet	128.8 M	15.48 G	96.68 ± 0.08	94.47± 0.07	97.73 ± 0.11	96.92 ± 0.14 *	94.43 ± 0.09	97.96 ± 0.16 *
ResNet-18	11.18 M	1.83 G	96.84 ± 0.06	94.38 ± 0.19	97.87 ± 0.11	97.14 ± 0.04 *	94.97 ± 0.22 *	98.06 ± 0.10 *
ResNet-50	23.51 M	4.15 G	97.05 ± 0.05	94.66 ± 0.03	98.05 ± 0.06	97.27 ± 0.04 *	95.07 ± 0.03 *	98.20 ± 0.06 *
ResNet-101	42.5 M	7.90 G	**97.28 ± 0.11**	95.06 ± 0.25	98.21 ± 0.09	97.39 ± 0.04 *	**95.38 ± 0.08** *	98.23 ± 0.02
OCTNet	1.86 M	1.16 G	96.73 ± 0.12	94.20 ± 0.13	97.79 ± 0.11	96.95 ± 0.10 *	94.45 ± 0.10 *	98.00 ± 0.03 *
8-layer	**1.80 M**	**0.69 G**	97.08 ± 0.04	94.81 ± 0.12	98.04 ± 0.04	97.25 ± 0.05 *	95.06 ± 0.07 *	98.17 ± 0.04 *
10-layer	1.96 M	0.93 G	97.27 ± 0.03	**95.13 ± 0.12**	98.17 ± 0.02	97.30 ± 0.06	95.23 ± 0.04	98.17 ± 0.05
12-layer	2.59 M	1.17 G	**97.28 ± 0.09**	95.00 ± 0.14	**98.23 ± 0.05**	**97.40 ± 0.04** *	**95.38 ± 0.14** *	**98.25 ± 0.05**

* indicates that the performance difference between multi-scale learning and single-scale learning is statistically significant of a *p*-value of 0.05.

**Table 11:** Performance of 12-layer SRN under various image sizes. The best results are highlighted in **bold**. ↑ denotes the higher, the better.

Learning Method	Image Size	Accuracy ↑	Sensitivity ↑	Specificity ↑
Single-scale	112×112	96.51 ± 0.07	93.86 ± 0.02	97.63 ± 0.10
	224 × 224	**97.28 ± 0.09**	**95.00 ± 0.14**	**98.23 ± 0.05**
	448 × 448	97.22 ± 0.04	94.88 ± 0.12	98.20 ± 0.07
Multi-scale	$112^{2}\;+\;224^{2}$	97.32 ± 0.04	95.27 ± 0.06 *	98.18 ± 0.04
	$224^{2}\;+\;448^{2}$	**97.40 ± 0.04** *	**95.38 ± 0.14** *	**98.25 ± 0.05**
	$112^{2}\;+\;448^{2}$	97.27 ± 0.05	95.02 ± 0.10 *	98.21 ± 0.03
	$112^{2}\;+\;224^{2}\;+\;448^{2}$	97.39 ± 0.01 *	95.37 ± 0.17 *	98.24 ± 0.06

* indicates that the performance difference between multi-scale learning and single-scale learning is statistically significant with a *p*-value of 0.05.

## Data Availability

Data available on request (The data used in the study maybe available depending on the corresponding authors and/or IRB’s decision).
